# Big data, qualitative style: a breadth-and-depth method for working with large amounts of secondary qualitative data

**DOI:** 10.1007/s11135-018-0757-y

**Published:** 2018-04-26

**Authors:** Emma Davidson, Rosalind Edwards, Lynn Jamieson, Susie Weller

**Affiliations:** 1 Centre for Research on Families and Relationships, University of Edinburgh, 23 Buccleuch Place, Edinburgh, EH3 9LN UK; 2 Sociology, Social Policy, Criminology, University of Southampton, Southampton, SO17 1BJ UK; 3 Centre for Research on Families and Relationships, University of Edinburgh, Chrystal Macmillan Building, 15a George Square, Edinburgh, EH8 9LD UK; 4 ESRC National Centre for Research Methods, University of Southampton, Southampton, SO17 1BJ UK

**Keywords:** Archived data, Big data, Big qual, Breadth-and-depth method, Qualitative analysis, Secondary analysis

## Abstract

Archival storage of data sets from qualitative studies presents opportunities for combining small-scale data sets for reuse/secondary analysis. In this paper, we outline our approach to combining multiple qualitative data sets and explain why working with a corpus of ‘big qual’ data is a worthwhile endeavour. We present a new approach that iteratively combines recursive surface thematic mapping and in-depth interpretive work. Our breadth-and-depth method involves a series of steps: (1) surveying archived data sets to create a new assemblage of data; (2) recursive surface thematic mapping in dialogue with (3) preliminary ‘test pit’ analysis, remapping and repetition of preliminary analysis; and (4) in-depth analysis of the type that is familiar to most qualitative researchers. In so doing, we show how qualitative researchers can conduct ‘big qual’ analysis while retaining the distinctive order of knowledge about social processes that is the hallmark of rigorous qualitative research, with its integrity of attention to nuanced context and detail.

## Introduction

Data sets from qualitative studies increasingly are stored in digital archives and available for reuse, notably in the UK but also more widely (Corti 2017). Examples of centralised and local repositories include the UK Data Archive, Finnish Data Service, Irish Qualitative Data Archive, Murray Research Centre (Harvard), and Wiener Institute for Social Science Data Documentation and Methods. As well as the opportunity for researchers to reanalyse a discrete data set, asking questions of it from different conceptual, substantive and analytic preoccupations to those employed by the original researcher/s, these archives present the possibility of conducting secondary data analysis across several, merged, qualitative studies. The case for what was referred to as ‘scaling up’ across multiple qualitative data sets saw light in a scoping paper produced for the UK’s Economic and Social Research Council by Jennifer Mason in 2002, which concluded: ‘perhaps the most significant opportunity offered to qualitative data is the possibility of “scaling up” through data sharing, to produce cross-contextual understandings and explanations’ (Mason 2002: 4). Mason recommended investment in qualitative studies, especially longitudinal, that were designed to provide data for secondary analysis. Longitudinal data were commended because of the particular opportunities afforded for understanding social change albeit also recognising the value of comparing cross-sectional data collected at different times. Following a review of qualitative data resources (Henwood and Lang 2003) and a feasibility discussion paper (Holland et al. 2006), one result was the ESRC Timescapes qualitative longitudinal study; an initiative that developed the specialist infrastructure for the sharing of qualitative longitudinal research (Neale and Bishop 2012; Neale et al. 2012). This study, described below, forms the backdrop for the development of our arguments about working with ‘big qual’ or large volumes of qualitative data.

The idea of working across existing multiple small-scale archived qualitative studies is relatively new. Mason identified the need for: ‘*appropriately qualitative ways* to “scale up” research resources currently generated through multiple small-scale studies, to fully exploit the massive potential that qualitative research offers for making cross-contextual generalisations’ (*ibid*: 3—our emphasis). Arguably this search for qualitative ways of dealing with large amounts of secondary data remains a challenge. It is one that we seek to address in this article. We do not, though, regard this as the one-way process implied in the term ‘scaling up’, from small scale to big, or from intensive to extensive levels. Rather, we present an approach that uses an assemblage of multiple qualitative data sets into a new corpus to enable analysis of issues beyond the foci of the constituent parts. Analysis is achieved by iteratively combining recursive surface thematic mapping and in-depth interpretive work; an approach that we refer to as the breadth-and-depth method. To aid discussion and practice of how we can enter ‘big qual’ data with qualitative integrity we will draw on several archaeologically-related metaphors.

The breadth-and-depth method emerges from our ongoing methodological project: ‘Working across qualitative longitudinal studies: a feasibility study looking at care and intimacy’ (ESRC 2015-2018, http://bigqlr.ncrm.ac.uk) which has a substantive focus on shifts in vocabularies and practices of care and intimacy over time by gender and age cohort. For the purposes of this article, we do not describe our findings or give more than minimal particulars of the data sets, because we wish to convey the applicability of the procedures to other research questions and other assemblages of data.^1^ Our project’s aim is to develop secondary analytic practice for working with multiple sets of in-depth temporal qualitative data to produce analyses that develop defensible methods of traversing both breadth horizontally and depth vertically. We are using existing material from six empirical projects, archived and made available digitally for re-use through *Timescapes* (ESRC, 2007–2012, www.timescapes.leeds.ac.uk). Despite some variation in substantive focus and method, the projects all traced personal and family relationships over time, each emphasising a different life-course phase, and are suitable for our project’s focus on care and intimacy practices. Our composite dataset comprises around 700 text files (transcripts, observations, field notes and diaries). Our goal is to illustrate a process that can be used in dealing with large volumes of pooled qualitative data; a volume that clearly exceeds the capacity of researchers to read all the data, yet remains true to the principles underpinning the conventional repertoire of techniques of qualitative data analysis.

The notion of big data is a backdrop both to the potential of working across multiple qualitative studies and to the ‘culture of uneasy suspicion’ surrounding qualitative secondary analysis (Mason 2007). Big data has emerged contentiously as the ‘new’ material that social researchers need to address (Burrows and Savage 2014). Its possibilities blur the boundaries of qualitative and quantitative research, as ‘big qualitative data’ invite analysis on a scale previously only usual in quantitative work with large digital datasets. The volume of textual data generated by qualitative researchers in any topic area is unlikely to rival the scale of ‘big’ volumes of textual data generated by social media, nonetheless, it may exceed the reading capacity of a typical project team. The idea of moving towards working with the totality of qualitative data in a topic area is one that qualitative researchers may view askance. ‘Why would you want to do that?’ is a comment we have received more than once from audiences of qualitative researchers when presenting our ideas about the feasibility of conducting secondary analysis across existing multiple small-scale archived qualitative longitudinal studies. In this paper, we review the emergence of big data before explaining why working across (relatively) large amounts of qualitative data is a worthwhile endeavour. In the rest of the paper we demonstrate a breadth-and-depth method for how qualitative researchers can work with ‘big qual’ data while retaining the distinctive order of knowledge about social processes that is the hallmark of rigorous qualitative research, with its integrity of attention to nuanced context and detail.

## The emergence of ‘big’ data

A host of computational processing tools and procedures have afforded social scientists the ability to manipulate information at a volume and speed never seen before. These technologies have made our social worlds more quantifiable and, in turn, offered new ways to make claims to the objectivity and accuracy of quantitative science (boyd and Crawford 2012: 667). Critics have hastened against sensibilities which position bigger volumes of data as offering ‘better’ or more accurate forms of knowledge (see for example boyd and Crawford 2012; McRobbie 2016). This viewpoint calls for the biases within big data to be acknowledged, and in particular, greater attention given to the context of its creation and use. The ‘context’ of big data relates to the social and cultural circumstances in which it was generated; how it was sourced and cleaned; and the approaches taken to analysis. The volume and procedures associated with big data creates the temptation to dispense with any role for theoretically or substantively driven analysis (Harford 2014: 187). This is perhaps particularly so when using data where context is absent or difficult to obtain (e.g. tweets and other social media). The consequences can be that context, and its associated meaning, is lost, misunderstood, or left unexplained (boyd and Crawford 2012; Andrejevic 2014; Lupton 2015). In contrast, Susan Halford and Mike Savage (2017) call for wider adoption of theoretically driven data assemblage in a style the authors call ‘symphonic’ because it interweaves data from multiple sources as mutually supporting themes of evidence.

There is also a risk that the prevailing discourse around big data as ‘better’ recreates the divide between quantitative and qualitative approaches and reasserts the superiority of the former. It implies that qualitative research has only a limited, or no role, in advancing the analytical practices that big data offers, and indeed many qualitative researchers seem prepared to concede this point. In spite of ever-reducing resources (Haiven and Khasnabish 2014; Hayfield et al. 2014) and the growing orthodoxy of mixed method approaches (Hesse-Biber 2010), committed qualitative researchers have continued to focus on small-scale quality. While the value of small-scale studies must be recognised, it is important that big data developments do not devalue qualitative research, or marginalise it to a small component of a larger mixed method study.

This article takes up approaches that blur disciplinary boundaries between computational sciences and social sciences. These do not see computational analysis as an “end in itself”, nor conventional qualitative data an ‘add on’. Rather we are advocating a form of analysis where approaches to computational text mining of large volumes of qualitative data sit alongside, and equal to, “deep data” research (Bruns 2013). Housley et al. (2014: 12) concur, stating that big data has the potential to make research “richer and more nuanced”. Such interdisciplinary meshing of approaches introduces a complementary approach where qualitative and quantitative techniques are in active dialogue, thereby challenging the “methodological anxieties” associated with big data (Savage 2015: 189). This point is important because there are potential advantages in the knowledge claims that qualitative researchers can generate through working with ‘big qual’ data.

## Beyond ‘scaling up’

The term ‘scaling up’ does not fully fit the iterative way of working with assemblages of data sets described here, although our ambition is to extend principles of qualitative research to the whole process. We are not ‘making large’ a process identical in structure to its smaller elements but using hybrid iterations of text mining and qualitative techniques that bring advantages. These are the scoping out of new research questions, drawing on the opportunities created for comparison and claims to generalisability. In our use of the word ‘assemblage’ we want to indicate that not only are data put together but they may be organised in new ways, for example, distributing project cases into categories that can be applied across projects (discussed below).

The type of assemblage that we are particularly concerned with involves researcher-generated digital material, such as interview transcripts and field notes, from multiple archived social science projects. While the techniques we describe here can be applied to other forms of meta-data tagged ‘big qual’ data, distinctly we are using archived qualitative data. Such material is an under-used resource with the advantages of associated documentation of methods and having passed thresholds of quality control. Secondary analysis of such data is less common than it is of archived large surveys. Qualitative researchers often complain that they ‘have not used all of their data’. This complaint expresses a belief that investing more of their own time in analysis would yield further value, rather than a plea for secondary analysis. Our focus is not deriving ‘more’ from the data of single studies but the gains from pooling data from multiple studies sharing the same broad topic. There can also be some concerns on the part of primary researchers about newcomer analysts as potentially blind to how data are shaped by the context of their collection and insensitive to the interests of originating research participants and researchers (Coltart et al. 2013). We believe it is possible to remain alert to such issues and add value to existing investments in qualitative research by ‘pooling’ data across studies without the consequential loss of distinction that might be implied by merging data sets together.

Such created assemblages offer opportunities for new research questions to be addressed making comparative use of differences between the studies. Information about the ‘context’ of each study (for example, specific focus, unit of analysis and sample characteristics, geographical and temporal location, researchers’ social science disciplines and approach to data collection or analysis) are metadata that can structure a comparative design. Researchers are then able to ask questions that could not be answered by individual projects and data analysis is enriched at each step. Some assemblages enable comparison across disciplinary differences, for example in research synthesis and systematic review (Campbell et al. 2006; Dubois and Gadde 2014; Sandelowski and Barroso 2006). While cross-disciplinary analytic approaches, such as the combination of psychoanalytically oriented analysis of biography with narrative analysis, life course perspectives or oral history accounts, are already advocated in discussions of mixed methods (Brannen and Moss 2012). We do not wish to repeat these discussions here but to note that, despite some exaggerated claims to the contrary in the literature on synthesis, they bring us back to the importance of attending to the context in which data are produced rather than seeing data as-if autonomous of its origins.

In the case of our own particular assemblage of data across projects, data are reorganised to serve our interest in gender and age cohort vocabularies and practices of care and intimacy. The information attached to transcripts about the birth date and gender of interviewees is used to reassemble the pooled data into age-cohorts of men and women in the new data assemblage. This enables us to move away from an analysis by project, and to undertake a comparison that addresses research questions about change over historical or biographical time in gendered vocabularies. For some projects, all participants were more or less the same age at the time of the study and the majority of cases consequently are allocated to one cohort. For projects involving more than one generation, however, cases are allocated to more than one cohort. The steps in analysis of differences between these gendered age cohorts are also informed by project-level metadata as well as the project-participant data on gender and year of birth. For example, consideration is given to how differences in the focus of projects influence the vocabularies used by researchers and research participants, the ways in which such differences then might complicate comparison by gendered age cohorts and lead to a misinformed account of social change over time.

Pooling data across projects may also enhance the possibilities of generalising from the data. Multiplying the number of small unrepresentative samples will never add up to a representative sample, and so there is no possibility of this type of claim to generalisability. However, increasing the diversity of samples and total number of research participants may strengthen claims about understanding how social processes work (see contributions to Baker and Edwards 2012). This is the type of claim to generalisability often made in textbook accounts of analysing qualitative research (Mason 2002; Lewis and Ritchie 2003; O’Reilly 2012). It is assumed that in-depth exploration that provides detailed understanding of how and why a particular process in social life unfolds in one way or another in one place and time helps understanding of similar processes in other places and times. Diversity, assembled through multiple distinctive small samples, softens the complaint that ‘unwarranted assumptions are made about the characteristics of the population of cases not yet studied’ (Seale 1999, 112). Pooling data may also enhance the possibilities of asking questions that can be tested against evidence and alternative argumentation. This is what Payne and Williams (2005) named as ‘moderatum generalisation’ and called for more of in their critique of qualitative researchers for failure to plan the possibility of evidence-based generalisation into research design. Explicit claims to such forms of generalisation are rare (Fairweather and Rinne 2012). New assemblages of data across projects can be created that use the possibilities of comparison to explore modest propositions, more explicitly addressing generalisability. In our case, for example, pooling data from multiple projects with a substantive focus on care and intimacy, reassembled into gendered age cohorts enables us to address claims about social change over time that point to convergence in men’s and women’s vocabularies and practices.

## Uses of metaphor: beyond mining and scaling up

Metaphors are a rhetorical semantic device that offer a framework to describe and structure, an imagery to facilitate our understanding.^2^ They transfer or translate one conceptual domain or coherent organisation of experience to another conceptual domain, to produce meaning by effective analogy that points up shared characteristics. Metaphors are common as part of everyday language and communication, political rhetoric and discussion, and so on (e.g. Charteris-Black 2016), but they may also be used to build and/or convey theories and models (Sewell 2010)—as in the use of a symphonic metaphor in the call to reorient big data analysis noted above (Halford and Savage 2017). Below we draw on some archaeological metaphors in putting across the notion of the data sets that comprise ‘big qual’ as a landscape, both as a whole vista to be mapped in its breadth and as containing interesting features to be dug into in more depth of detail—our breadth-and-depth method. In other words, we can combine extensive coverage with intensive illumination.

Several authors have used a metaphor of archaeology to capture and illuminate data analysis that addresses change over time involving both breadth and depth. Zimbra et al. (2010) coin the term ‘cyber-archaeology’ to describe their approach to online social movement research, which they assert overcomes many of the issues of scale and complexity facing social research in broad and longitudinal research on virtual communities. This moves from identification of online domains of interest, an automated collection and classification of a lexicon of ‘cyber-artefacts’, to social network analysis and visualisation. In turn, Clive Seale and Jonathan Charteris-Black worked with a corpus of over 1000 interview transcripts and undertook a matched comparison using a sub-sample of 102 interviews selected by age and gender in order to explore narrations of the experience of cancer, and use the metaphor of an ‘aerial archaeologist’ to refer to their first step analysis looking at key word patterns, which then ‘descends’ into qualitative analysis:

Keyword analysis is like an aerial view of a landscape, whose undulations and patterns of vegetation growth reflect the outline of ancient buildings, only possible to see from the air. At this point, the ‘aerial archaeologist’ descends to ground level and starts to dig. (2010: 537)

Like these authors, the methods we are using include a stage of mapping using techniques that are sometimes referred to as ‘data mining’. This use of ‘mining’ is not, however, a helpful metaphor for this stage of the process because it suggests deep digging. For a qualitative researcher the ‘depth’ in the metaphor of vertical digging is associated with tracing meanings and processes in the complex specificity of relevant framing contexts. This is a very different kind of approach to types of data mining that involve algorithm-driven sifting and sorting words in search of associations and that treats text as bags of disassociated words or order strings of more or less proximate words. In our view, this kind of so-called mining is about breadth, surface thematic mapping and not depth but can be deployed as an integral part of a series of iterative steps that move towards combining breadth and depth.

The process of iteratively combining breadth and depth to assemble interpretive meaning involves a series of steps: (1) overview survey of archived data sets and then creating a new assemblage of data that become the corpus on which the next steps are performed; (2) recursive surface thematic mapping in dialogue with (3) preliminary analysis, remapping and repetition of preliminary analysis; and (4) in-depth analysis of the type that is familiar to most qualitative researchers on materials identified by previous steps. We can describe these steps by metaphorically transposing from the conceptual domain of analysing across qualitative data sets to methods of archaeological excavation (e.g. Drewett 2011) to evoke the idea of moving between breadth and depth while retaining the integrity of a contextualized and detailed qualitative approach.

## Steps in our breadth-and-depth method

### Overview survey of archived qualitative data and construction of a corpus

The reconnaissance of practically accessible archived, and in many cases digitised, academic data sets to identify an appropriate territory of study (i.e. assemblage of data sets) that might be suitable for the intended analysis is akin to an archaeological aerial survey. What is practically accessible is contingent on the skills and energies of the researcher, as well as the state of the art of archived data. Archived data typically comes with a meta narrative in the language of the national context that records the aim of the study, disciplinary approach and method. Meta data—such as type of data, date of collection and the socio-demographic characteristics of research participants—are typically attached to items within archived data sets. Scrutiny of such narratives and associated lists of meta data provide a precursory understanding of the nature, quality, and suitability of data sets for inclusion in the assemblage that will become the new corpus according to the ‘fit’, as Hammersley (2010) describes, to the research endeavour.

Indeed, the process is likely to be familiar to those working in a range of disciplines where the use/re-use of archived materials is commonplace to address research questions (for examples, see Luff et al. 2015). When reviewing potential sources for inclusion in the assembled new corpus, the researchers’ questions set the criteria, the topic of study, and geographical or linguistic context to be sought. The initial criteria may be broad ranging or narrow, locating data sets on wide topic areas or focused on identifying one or a few very specific substantive issues of interest. Considerations may include the geographical focus, theoretical perspective, epistemological stance, sample characteristics, and units of analysis. During this initial overview step, decisions are made about the inclusion or exclusion from the newly assembled corpus and the uses of meta data to structure files within the new assemblage.

Surveying the contextual material and meta data associated with the Timescapes data sets for our particular study, for example, provided us with a sense of the scope and nature of these data including data format and volume, research tools used, the substantive emphasis (also given by published papers and data collection instruments such as interview schedules), and an understanding of the temporal rhythm of data collection including the spacing of each wave of data. In short, we documented the ‘who’, ‘what’, ‘where’, ‘when’ and ‘how’ of the original research endeavours. The process also revealed anomalies in the landscape such as gaps in contextual material or unusual, misplaced or incorrectly labelled or categorised files. We compiled our own database of files across Timescapes projects using our audit of the meta- and contextual data and to retrieve files/folders appropriate to our particular analytic focus on care and intimacy across gender and age cohort. Once complete the constituent data became one entity—the corpus for our project. In this assemblage data were organised by age and gender cohorts but retained their link to each of the original projects in their meta data.

### Recursive surface ‘thematic’ mapping

Geophysical surveying is an approach used by archaeologists to gain insight into a field of study without disturbing the landscape. The patterning of landscape features can be recorded, mapped and visualised from the surface to detect areas of interest for further investigation. In the context of analysing large volumes of qualitative data, the equivalent processes of mapping the landscape involves working back and forward between computer-aided surface ‘thematic’ mapping across the corpus^3^ and reading of short extracts around samples of ‘themes’ to check their relevance. This is a recursive process likely to involve multiple iterations as readings will sometimes reveal text that is ambiguous in meaning or tangential to the research questions. This would, result in elimination of the ‘theme’ and a return to the mapping process.

Theme is placed in parenthesis above because the surface mapping techniques used are not typically sufficiently refined to identify the coherent cluster of meanings and sense making that might be conjured up by the term. There are multiple computer-aided means of taking more or less sophisticated steps towards thematic mapping including use of word frequency, searches for themes based on word proximity and association, and searches matching words to pre-given dictionaries, such as positive and negative feeling words. One starting point, for example, is a keyword analysis and identification of keyness as indicative of potential themes—techniques widely used in corpus and linguistic studies (see Jockers 2014). A keyword, at this stage of the analysis, is defined as a word that occurs with unusual frequency in a given text. This does not equate to words that are numerically greater, but are unusually high in comparison to a reference corpus of some kind (Scott 1997). Each study would need to specify the basis of the ‘norm’ against which keyness can be calculated. Adopting a realist methodology, for example, Wendy Olsen and Jamie Morgan (2010) used Computer Assisted Qualitative Data Analysis (CAQDAS) to compare their dataset to the British National Corpus as a means of identifying the significance of particular words. Keywords were grouped into ‘discourses’ which were studied in context. Rather than use a general reference corpus, Seale and Charteris-Black (2010) compared texts with each other using text-mining software. Here, the gendered experiences of illness were explored by examining the relative ‘keyness’ of keywords. These were grouped into meaningful clusters with shared semantic meanings.

For our own project, the focus has been upon identifying keywords, with gender and age cohort by groupings of birth year as the source of comparison. We were able to identify keywords associated with care and intimacy, and their relative keyness within gender and cohort by grouped years of birth. Rather than searching for particular words that researchers believe might have salience, this approach allowed an openness to the data, and for it to reveal aspects of care and intimacy that otherwise might have been missed or overlooked.

### Preliminary analysis

Key words or ‘themes’ identified by the mapping process can then be sampled for further preliminary examination. In archaeological metaphor terms this is akin to digging shallow test pits, where the digging is only deep enough to show whether anything of interest is present in the data extract being examined. When starting from clearly defined research questions, certain emergent key words or themes may be substantively or theoretically more attention-grabbing and seem like the obvious starting point. Nevertheless, samples of both the more and the less promising should be subjected to preliminary analysis before deciding where to dig deeper before taking the process to the more conventional strategies familiar to qualitative researchers of reading whole texts (see Sect. 5.4).

Even the preliminary analysis is conducted with mindfulness of the context in which extracts were generated. The landscape of possibilities is viewed with knowledge of the metadata attached to the text, and the source project. The preliminary analysis involves reading relatively short extracts (we usually use around 200 words), enough to encompass several full sentences and provide a clear sense of whether or not the content speaks to the researcher’s research questions. It is important not to be drawn into wider or deeper reading at this stage since multiple sites must be given this level of preliminary examination in order to justify where to undertake deeper analysis. When a substantial proportion of samples of a key word or ‘theme’ prove ambiguous or tangential, it must then be eliminated and the researcher returns to the mapping to try again.

How many samples should be subjected to preliminary analysis to be enough provokes the same ‘it depends’ answer as ‘how many qualitative interviews is enough’ (Baker and Edwards 2012). In choosing a number of samples, consideration should be given to the different logics of sampling currently operating within qualitative research: theoretical, purposive and realist (Emmel 2013) as alternative variants on a proportional approach taking every nth case across the multiple possibilities among indicators of keyness or themes. The logic of sampling will be shaped by attention to the research questions and research design. A stage of preliminary analysis of samples should not proceed without considering whether the sampling approach is problematic if different levels of attention are given to categories that are the basis of comparison. For example, in our own study using age cohort and gender differences in vocabularies and practices of care and intimacy, we are exploring the claim that gender difference in understandings of these practices reduces down generations. If our key word mapping finds fewer intimacy words among older men, it would be problematic to translate this into choosing fewer samples for preliminary analysis.

The total number of samples will also be set pragmatically by time available, informed by time taken to reach this stage and the need to preserve a greater period for the next. There is also the possibility that some or many samples may prove to be of no analytical value and a new set of samples chosen on the basis of the next most promising keyness or theme possibility. Indeed, it might be necessary to go back to re-run the mapping process and look again, having dispensed with attention to the particular barren features.

### In-depth interpretive analysis

The next step moves from examining extracts of data to working with whole cases. In-depth interpretive analysis, the ‘deep excavation’ of cases from ‘big qual’, is a process that the majority of qualitative researchers will recognise, value and be comfortable with as a ‘thinking, reflexive, practitioner’ (Mason 1996: 8). It is an immersion in data at a scale that qualitative researchers feel uses the strengths of qualitative analysis; that is, in being sensitive to changing context, multi-layered complexity and rich detail to represent intricate social realities and produce nuanced social explanations. This is the stage in working with ‘big qual’ that brings depth back into conversation with breadth.

The question of ‘how many is enough’ surfaces again for cases to excavate. As with sampling for preliminary analysis, the logic of selection will be shaped by the research questions, the research design, and the different points of further interest thrown up by the thematic analytic ‘digging’. The external empirical parameters of the various data sets pooled in the corpus also mean that it is important to have an eye to selection of pertinent cases from across the various data sets. If all cases identified are from one of the constituent data sets alone, for example, this should raise questions about the fit between data sets and the recursive thematic surface mapping process.

There is a diversity of qualitative analytic strategies and in-depth techniques that illuminate, variously, social meanings, subjectivities, activities, processes, constructions and discourses. A representative textbook addressing qualitative data analysis, for example, includes (amongst others): thematic analysis, frame analysis, event analysis, grounded theory, narrative analysis, conversation analysis, discourse analysis, visual analysis, and semiotic analysis (Grbich 2007). Which analytic technique, or combination of techniques, and focus are adopted is determined by the researchers’ epistemological stance, conceptual approach, substantive concerns, and the pragmatics of the form/s of data. Which units of analysis or cases are selected for in-depth analysis from the multiple archived small-scale qualitative studies that form the corpus also is dependent on the previous processes in interaction with the epistemological and theoretical standpoint, and the substantive research topic and questions, as we note further below.

We need to acknowledge though, that working across multiple small-scale data sets can raise knotty questions about what constitutes a case. Different data sets may speak to the same set of intellectual and substantive puzzles but may have collected data from different sorts of sources in different forms (identified through the aerial survey). In the Timescapes study, for example, while all the projects were concerned with aspects of how personal and family relationships develop over time, they each produced and archived a variety of forms of qualitative longitudinal data for (re)analysis. These materials were based on repeat interviews with the same individual, and/or with a group of connected individuals interviewed individually and/or collectively. The data collection involved the production of visual materials such as network maps and photographs, and written materials such as participant diaries and researcher observations. Additionally, the researcher who contacted and interviewed participants for a project was not necessarily the same person every time. With such differences of data sources across a ‘big qual’ corpus, secondary researchers need to consider whether a case is an individual research participant, a set of participants, the research encounter, a period of time, a geographical location, or an institution or organisation, and covers all forms of material. The guiding mechanism for decisions about what constitutes a case lies in the rubbing together of the ‘internal’ intellectual purpose of the secondary researcher’s project, and the ‘external’ empirical parameters of the various sets of qualitative data available to them to create the corpus to pursue that knowledge (Emmel 2013).

As well as a reflexive interplay between intellectual and empirical contexts in deciding the constitution of a case then, in our iteratively combined breadth-and-depth method, case selection is rooted in the interaction between the potential characteristics of comparison identified through auditing the meta data, the surface mapping of keyness or ‘themes’ and preliminary examination of sampled extracts that confirms points of salience for further in-depth analysis. In our own study, for example, our interest in comparisons of intimacy and care by gender and age cohort led us to focus on individuals and their interview transcripts as our cases. Our points for in-depth investigation were chosen by clusterings and configurations of keyness identified through mapping and preliminary analysis interacting with our comparative interest in age cohort and gender. And our interest in time leads us to turn to means of in-depth analysis that have a temporal focus, such as historical and biographical timelines, and biographical analysis (e.g. Chamberlayne et al. 2000), thematic analysis of recurrent stories (e.g. Braun and Clarke 2006), and trajectories and turning points (e.g. Abbott 2001). Working across cases then enables a deep comparison that takes account of lives in context.

## Conclusions

With the changing context of social research comes new opportunities for qualitative researchers. As we have discussed, the sharing of data is widely recognised by major research funding bodies in the U.K. as vital to accountability and transparency and for some, such as the ESRC, it is a contractual requirement (Mauthner 2012). Moreover, the increasing significance and influence of big data, which has, to date, generally concerned large-scale quantitative datasets, highlights potentials for qualitative researchers to enhance further the value of existing investments. Robin Smith (2014: 184) has voiced concern about the potential for big data and its analytical techniques to separate methods from methodology and discipline. Our approach is precisely the opposite. Computerised text mining has facilitated something beyond ‘scaling up’, making the shift between breadth and depth more transparent, enabling us to move across disciplinary and epistemological perspectives and introduce cross-contextual generalisations.

We contend that working with assemblages of qualitative data sets, from multiple longitudinal or short-term studies, is a worthy endeavour in that it offers the possibility of enhancing the richness of research. By working across pooled and synthesised data sets, new substantive insights into social processes are gained. We see new assemblages of data as a means of further strengthening the use of comparison to aid generalisability. We also argue that working with a corpus of ‘big qual’ goes beyond other forms of research that synthesise multiple studies, such as systematic reviews, in that researchers re-visit the datasets rather than pulling together the findings of others as they have been reported. We are also mindful not to disregard small qualitative studies and consider our suggested approach as a way of further bringing out the value of these.

Our aim has been to outline a systematic and rigorous approach to ‘big qual’ analysis in a manner that maintains the integrity of qualitative work, pays attention to the context and richness of such research, and provides the means of handling large volumes of detailed data. As a result, we have proposed an iterative process to demonstrate the complex reality of how qualitative researchers can deal with these large volumes/multiple datasets. The advocated approach iteratively combines recursive surface ‘thematic’ mapping and in-depth interpretive analysis in order to achieve a form of analysing large amounts of archived qualitative data that enables the researcher to address new research questions beyond ‘scaling up’ primary analysis.

There are caveats to our approach of course, especially of time and resources. Pragmatically, while the surface ‘thematic’ mapping and preliminary analysis stage of our breadth-and-depth process can be achieved in much the same time using CAQDAS whatever the size of the corpus, clearly the other stages in the process are likely to be time- and resource-consuming, especially if researchers are dealing with visual as well as textual material. Bringing different data sets with their variable meta data dimensions, original researcher practices, and formatting into a common framework, can raise intellectual challenges too. For example, as discussed above, the constitution of generational cohorts was specifically constructed for our project and cut across the separate projects comprising the corpus, requiring dialogue between the theoretical and the empirical. These issues aside, we believe that we have a convincing answer to the sceptical qualitative question of ‘why we would want to do that’, and have here provided a guide to how researchers can conduct secondary analysis using a ‘big qual’ corpus of existing multiple small-scale studies while retaining qualitative integrity.

## Footnotes

1 More information about the Timescapes study, and access to its constituent data sets, is available at http://www.timescapes.leeds.ac.uk/archive/.

2 We also acknowledge that metaphor can ‘cast shadows’ and have the effect of shutting down on other meanings or misdirecting attention, as much as ‘shedding light’ (Carpenter 2008).

3 We acknowledge that we are primarily referring to text files such as transcripts, notes and diaries.
